# Supplementary material for: A deep learning-based stripe self-correction method for stitched microscopic images
Source: Nat Commun. 2023 Sep 5;14:5393. doi: 10.1038/s41467-023-41165-1 (PMC10480181; doi:10.1038/s41467-023-41165-1)
Supplement: Supplementary file 1 — Supplementary Information file [file 41467_2023_41165_MOESM1_ESM.pdf]

Supplementary Information

A deep learning-based stripe self-correction method for stitched microscopic images

Shu Wang[1, 2, 3, 9], Xiaoxiang Liu[2, 9], Yueying Li[1, 9], Xinquan Sun[1], Qi Li[2], Yinhua She[2], Yixuan Xu[1], Xingxin Huang[3], Ruolan Lin[4], Deyong Kang[5], Xingfu Wang[6], Haohua Tu[7, 8], Wenxi Liu[2, *], Feng Huang[1, *], Jianxin Chen[3, *]

[1]College of Mechanical Engineering and Automation, Fuzhou University, Fuzhou 350108, China.

[2]College of Computer and Data Science, Fuzhou University, Fuzhou 350108, China.

[3]Key Laboratory of OptoElectronic Science and Technology for Medicine of Ministry of Education, Fujian Provincial Key Laboratory of Photonics Technology, Fujian Normal University, Fuzhou 350007, China.

[4]Department of Radiology, Fujian Medical University Union Hospital, Fuzhou 350001, China.

[5]Department of Pathology, Fujian Medical University Union Hospital, Fuzhou 350001, China.

[6]Department of Pathology, The First Affiliated Hospital of Fujian Medical University, Fuzhou 350005, China.

[7]Beckman Institute for Advanced Science and Technology, University of Illinois at Urbana-Champaign, Urbana, IL, 61801, USA.

[8]Department of Electrical and Computer Engineering, University of Illinois at Urbana-Champaign, Urbana, IL, 61801, USA.

[9]These authors contributed equally: Shu Wang, Xiaoxiang Liu, Yueying Li.

[*]Correspondence: Wenxi Liu (wenxiliu@fzu.edu.cn); Feng Huang (huangf@fzu.edu.cn); Jianxin Chen (chenjianxin@fjnu.edu.cn).

Table of contents

Supplementary Note 1: Procedure of the artifact and stripe synthesis
Supplementary Note 2: Reasons and limitations of using deep learning comparison methods
Supplementary Note 3: User study
Supplementary Note 4: Ablation study
Supplementary Note 5: Brief introduction to the comparison methods

Supplementary Figure 1: Representative sampling cases of stripe and artifact
Supplementary Figure 2: Other representative stripe correction results
Supplementary Figure 3: Unbiased signal estimation across intensity levels of heterogeneous images
Supplementary Figure 4: Detailed correction results of imprecise prior stitched information
Supplementary Figure 5: SSCOR has tolerance towards the imprecise user-defined abnormal region for bubble-like artifacts
Supplementary Figure 6: Pipeline of the stripe and artifact synthesis
Supplementary Figure 7: Restoration results of real artifacts in the MPM images
Supplementary Figure 8: User study results based on the background of participants
Supplementary Figure 9: Additional representative cell classification results based on SSCOR-corrected images
Supplementary Figure 10: Representative correction results of synthetic stripes on H&E images
Supplementary Figure 11: Cell counting results on bubble artifact and stripe images
Supplementary Figure 12: Accuracy of automatic counting compared with manual counting
Supplementary Figure 13: Schematic diagram of typical stripes and artifacts
Supplementary Figure 14: Visualization of network architecture
Supplementary Figure 15: Model performance versus the training epochs with random initialization.
Supplementary Figure 16: The effect of sampling step size on model performance.
Supplementary Figure 17: Illustration of the local-to-global strategy
Supplementary Figure 18: Ablation study

Supplementary Table 1: User study results of MPM and fluorescence datasets with stripes
Supplementary Table 2: User study results of SRS datasets with synthetic stripes and artifacts
Supplementary Table 3: Requirements of different methods for training images
Supplementary Table 4: Description of stripe and artifact types and the corresponding sampling strategy
Supplementary Table 5: Comparison of the microscopic datasets
Supplementary Table 6: Proximity sampling on representative cases

Supplementary Notes

Supplementary Note 1: Procedure of the artifact and stripe synthesis

To precisely assess the restoration quality for the images with three types of artifacts, the clean SRS images are employed as the base images and ground truth, and different artifact masks are combined with them to mimic the images contaminated by artifacts. As follows, the details of generating stripes and artifacts are explained, respectively.

In practice, the stripes and special artifacts may exist in the stitched images at the same time. The process of various artifact synthesis can be formally defined as follows. Specifically, for the stripe, bubble, and out-of-focus artifacts, the synthesis process can be formulated as:

$$I_S = I_R * (1-\alpha) * I_M, \quad (1)$$

where the synthetic mask I_M, each pixel of which has an intensity value between zero and one, is combined with the region of raw image I_R to acquire the synthetic result I_S. α denotes the balancing factor for the masking impact of stripe and the three artifacts. The larger the value, the greater the effect of the mask, and vice versa. By altering the factor, diverse synthetic images can be obtained.

Stripe artifacts. To combine non-uniform stripes with stripe-free stitched images, the mean intensity of all the raw tiles from a stitched MPM image is calculated and normalized, which serves as shading-mask I_M. Then, we use each tile of a clean SRS image as I_R, and combine it with the mask I_M to produce the synthesized image I_S. By adjusting the balancing factor α in Equation (1), the diverse shading-masks I_M are acquired. Finally, the non-uniform stripe image is generated by stitching these synthesized tiles I_S.

Bubble-like artifacts. The bubble-like mask I_M is empirically designed to maximally imitate the real bubble-like artifacts in MPM images (Supplementary Fig. 7). In specific, to simulate the

non-uniform pattern inside bubble artifacts, the intensity attenuation of the mask I_M is manipulated by a Gaussian filter that smooths the pixel values around each center pixel. Thereafter, the mask I_M is combined with a random region I_R of the clean SRS image.

Out-of-focus artifacts. According to previous imaging experience and the real out-of-focus images, the out-of-focus artifacts commonly exists in the four corners of stitched images. Therefore, we empirically generate the mask of out-of-focus artifact I_M. The intensity of I_M is set to be faded in periphery, modeled by a Gaussian filter, to imitate the uneven out-of-focus affect. Then, we use the corner of clean SRS image as I_R, which will combine with I_M to produce the synthesized image I_S.

Scanning fringe artifacts. The nonlinear optical images often exhibit strong scanning fringe artifact (SFA) resulting from the fast galvo-resonant (GR) scanning system[1]. According to the reference that appears SFA[1], we observe that SFA often occurs in the blue channel with weak signal areas, due to the low efficiency of nonlinear process. Thus, we employ the blue channel of human ovarian carcinomas images as synthetic mask I_M to generate scanning fringe image I_S. The synthesis process can be formulated as:

$$I_S = I_R + \beta * I_M * 255, \tag{2}$$

where β refers to the balancing factor similar to α. In order to simulate SFA realistically, we define it as additive noise, and simply integrate I_M and I_R through addition operation. We add the mask I_M to the clean SRS images I_R and thus synthesize scanning fringe artifacts on the partial region of the raw image.

Supplementary Note 2: Reasons and limitations of using deep learning comparison methods

In the artifact removal experiments, we employ several deep learning-based unsupervised methods for comparison, according to the unsupervised learning settings of our task. As follows, we explain the reason of employing these methods and their respective limitations for our task.

ZeroDCE[2] was used in the experiments for removing the out-of-focus artifacts. Since the out-of-focus regions have lower intensity than the other regions in the stitched images, it can be removed by enhancing the brightness of the artifact regions to some extent. We tried several state-of-the-art unsupervised low-light enhancement methods including RUAS[3], SCI[4] and ZeroDCE[2] to explore this feasibility. Among these methods, ZeroDCE achieves the optimal performance. However, based on our observation, there is a large domain gap between the natural image domain and the microscopic image domain in texture contrast and resolution, which makes it difficult to adapt these methods on the task of out-of-focus artifact removal for the microscopic images.

Mask-ShadowGAN[5] was involved in the experiments for removing the bubble artifacts. Since the bubble artifacts appear to be similar to the soft shadow in natural images, we employ the state-of-the-art unsupervised shadow removal methods, Mask-ShadowGAN[5] and LG-ShadowNet[6], to remove bubble artifacts. Mask-ShadowGAN shows respectable performance. But, it treats shadow in the form of binary mask, so it excels at handing hard (uniform) shadow rather than soft (non-uniform) shadow. Thus, it does not perform well on the task of bubble artifact removal.

Neighbor2Neighbor[7] served as a representative image denoising method in the experiments for removing the scanning fringe artifacts. SFA can be considered as a special type of noise from the imaging system. Thus, we use the off-the-shelf image de-noising method, Neighbor2Neighbor[7] and Self2Self[8], to deal with the noise in scanning fringe artifacts. However, the noise pattern of SFA is significantly different from natural noises, so they fail to recover the original tissue signal from SFA.

Supplementary Note 3: User study

Subjective evaluation is a reliable method for assessing the quality of biomedical images, taking into account the human perception of the images. To further evaluate the effectiveness of different correction methods, an anonymous online survey was conducted with 50 participants from five different backgrounds, including computer vision, pathology, biophotonics, optics, and precision instruments. The survey included the test images of two types: (1) multiphoton microscopy (MPM) and fluorescence[9] datasets with stripes, totaling 305 images and (2) stimulated Raman scattering (SRS)[10] datasets with synthetic stripes and artifacts, totaling 120 images. To fully compare the correction effect, the images for user study included not only the global images but also the local images. Each set of user study images included the input image as well as the corresponding images corrected by the proposed SSCOR and the comparison methods (BaSiC[11], CIDRE[12], ZEN[13], ZeroDCE[2], N2N[7], and Mask-ShadowGAN[5]). The images were randomly assigned to the participants who were asked to rate a total of 26 sets of images on a severity scale from 0 to 10 according to the presence of stripes and artifacts in each image. The scoring criteria are as follows:

(a) 0-2, worst, severe stripes and artifacts, significantly interfering with the observation.

(b) 2-4, worse than average, heavy stripes and artifacts, interfering with the observation.

(c) 4-6, average, mild stripes and artifacts, slightly interfering with the observation.

(d) 6-8, better than average, slight stripes and artifacts, not affecting observation.

(e) 8-10, best, almost no stripes and artifacts.

The image sets were randomly displayed, and the participants were allowed to return and change their previous rating until submission. The results of the user study were presented in Supplementary Table 1 and 2. The study showed that SSCOR significantly outperformed other methods for stripe correction and artifact removal. In addition, we visualized the user study results with regards to the background of the participants in Supplementary Fig. 8, implying the significant advantages of SSCOR in each discipline.

Supplementary Note 4: Ablation study

Ablation studies were conducted on the proposed schemes, including proximity sampling, reciprocal training, and local-to-global correction, in order to demonstrate the reason for the model design.

Proximity sampling

To study the effectiveness of proximity sampling, we compare it with central sampling and random sampling strategies. For all of these sampling strategies, the patches on stripes are regarded as anomaly patches. The main difference rests in the way of sampling normal patches. The central sampling strategy utilizes the central patch of the tile as the normal patch corresponding to the closest anomaly patch, while the random sampling strategy randomly selects the patches from the regions off stripes over the entire stitched image. As illustrated in Supplementary Fig. 18a, the proximity sample strategy shows superior performance over the other two strategies in the stripe correction and signal restoration.

Next, the dimension of the sampled patches is studied, which depends on the width of stripe and the size of the image tiles. Empirically, it should be smaller than image tile and slightly wider than stripes, in order to enable SSCOR sample the proper normal patches. We compare the stripe removal results with different sizes of patch in Supplementary Fig. 18b. Considering the stripe size in most stitched images, we empirically set patch size as 256 as the default setting which obtains the better de-stripe results.

Adversarial reciprocal-training

In the adversarial reciprocal training scheme of SSCOR, the stripe synthesis network adds the shadings to the corrected patches in order to preserve image content, since the consistency between the original patches and the synthesized ones serves as effective constrain for stripe correction. We conduct visual comparison experiments to verify the performance with and without stripe synthesis network. As shown in Supplementary Fig. 18c, without stripe synthesis network, there still remains

the traces of stripes in the corrected image.

Local-to-global Correction

As the final stage of SSCOR workflow, local-to-global correction consists of two steps, i.e., local correction and global merging. First, the whole-slide images are partitioned into overlapping local patches in the sliding-window manner and fed into the stripe correction network to obtain corrected patches, which is the local correction step. Next, all the corrected patches are merged by computing the average of overlapped region between adjacent patches. The merging procedure yields a whole image with the same resolution as the original stitched image, which is the global merging step. This integration of local and global steps enables the suppression of stripes and fosters a smoother texture alignment between corrected patches.

To verify our overlapping local correction strategy, we compared it with the other two strategies, stripe-only local correction and non-overlapping local correction, on an MPM image with horizontal stripes and out-of-focus artifacts, where the out-of-focus artifacts exist in the upper left corner (Supplementary Fig. 18d). As observed, stripe-only local correction is able to suppress the shading of stripes, but cannot completely correct the out-of-focus areas beyond the stripes. As for non-overlapping local correction, the corrected result may exist stripe residues. To sum up, overlapping local correction obtains the most significant correction results (Supplementary Fig. 18d).

Supplementary Note 5: Brief introduction to the comparison methods

The comparison methods used in the experiments are introduced in the following.

As a retrospective method, CIDRE[12] operates on a set of image tiles corrupted by illumination distortion that are acquired under consistent conditions. It estimates shading distortion parameters using regularized energy minimization to correct intensity distributions.

BaSiC[11] is another retrospective method for background and shading correction of image tiles, based on a sparse and low-rank decomposition. It imposes sparse constraints on the

Fourier-transformed shading model to enforce a good shading correction.

ZEN[13] is a commercial software from Zeiss and it performs shading correction based on a shading reference image generated from image tiles. ZEN requires a raw image with meta information. Moreover, it demands over 300 tiles per image for a good reference image.

ZeroDCE[2] proposes a light-weight deep network for low-light image enhancement. It coverts this task into an image-specific curve estimation problem, which uses image as input, curve as output. The curves perform pixel-wise adjustment on the input images to get enhanced images. It can be trained end-to-end without any reference image by setting a series of non-reference losses, which implicitly evaluate the quality of output image.

Mask-ShadowGAN[5] presents a mask-guided generative adversarial network for shadow removal from unpaired training data. It models the relationship between shadow and shadow-free images by their difference, and uses the shadow mask (binary image) to represent the difference. The network learns to generate a shadow-free image and then produces a shadow image as guided by the shadow mask. By formulating the consistency constraints to obtain shadow images and learns to remove shadows.

Neighbor2Neighbor[7] is a self-supervised image denoising framework with only noisy images. This approach generates sub-sampled paired images by random neighbor sub-samplers from noisy images. Denoise one of the sub-sample image, and construct a reconstruction loss with the other one. Then denoise the original noisy image, and derive sub-sampled pair by using the same neighbor sub-sampler to calculate the regularization term.

Supplementary Figures

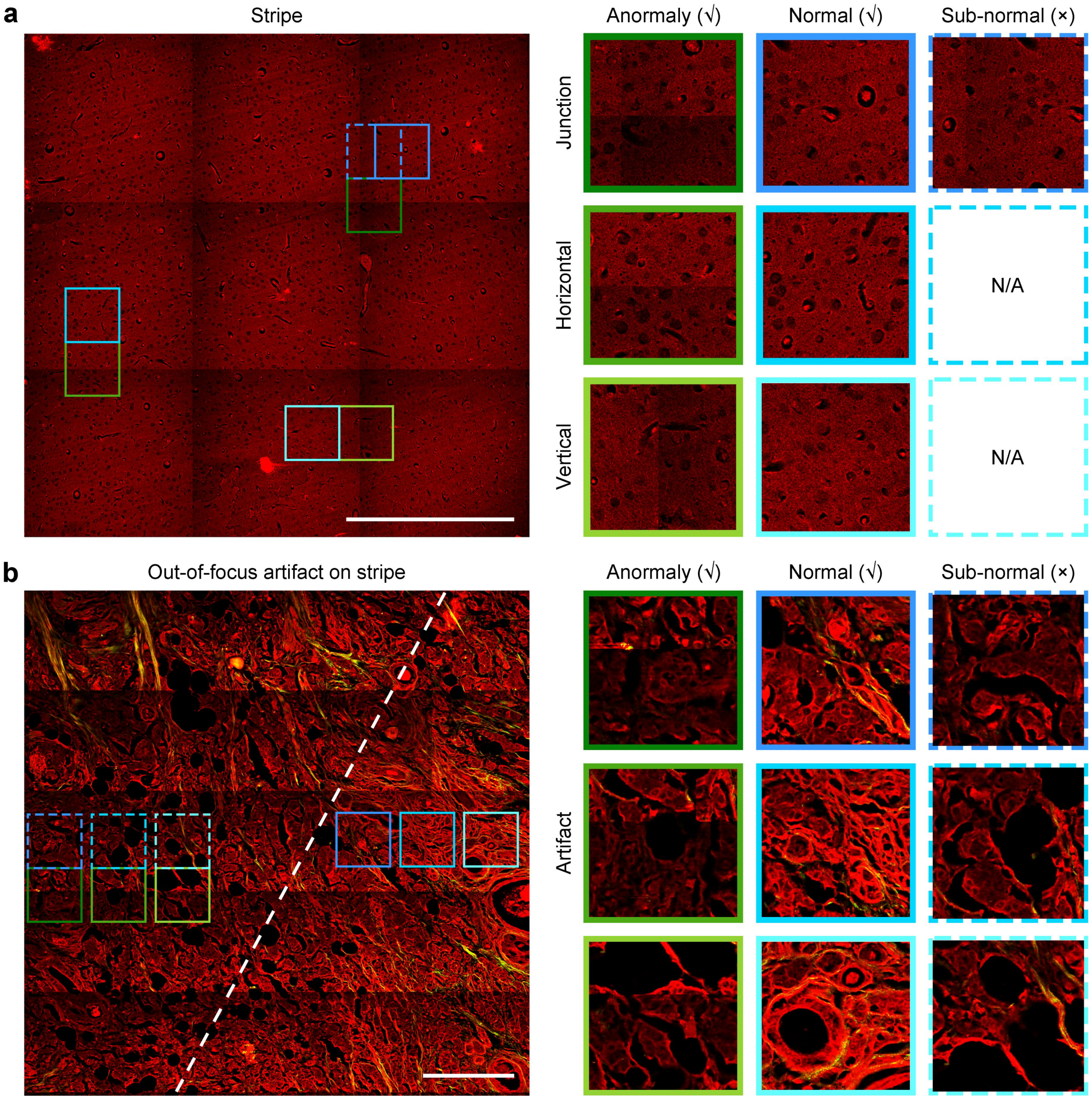

Supplementary Figure 1. Representative sampling examples with regards to stripe and artifacts. a) In the given stitched stripe image, the anomaly patches (green boxes) on stripes and the corresponding normal patches (blue boxes) in proximity are sampled and paired up for training. In concrete, the anomaly patches on the horizontal (or vertical) stripes pair with the nearest normal patches, i.e., the ones off the stripes along the vertical (or horizontal) direction. Note that, for the anomaly patch on the junction of the vertical and horizontal stripes, the nearest patches off the junction area along the vertical or horizontal direction (blue dashed boxes) are also affected by the stripes, namely sub-normal patches, and thereby they cannot be treated as normal patches for training. Thus, for the anomaly patches resting on the junction, the nearest diagonal patches are chosen as the corresponding normal patch, instead of choosing the sub-normal patches. Not applicable (N/A) denotes the absence of the corresponding image. b) For the image with both stripes and special artifacts (in addition to the stripes covering the entire figure, it contains the out-of-focus artifacts on the left side of the dotted line), the sampled anomaly patches (green boxes) from artifacts and the corresponding normal patches (blue boxes) constitute the unpaired training data. The nearest patches off the stripes along the vertical (horizontal) direction affected by the artifacts are also considered as sub-normal patches (blue dashed boxes). Likewise, these sub-normal patches cannot be used in training. Therefore, SSCOR samples the patches from the regions with out-of-focus artifacts as anomaly patches, meanwhile sampling the normal patches in the remaining areas without artifact. Scale bars in a) and b): 200 µm.

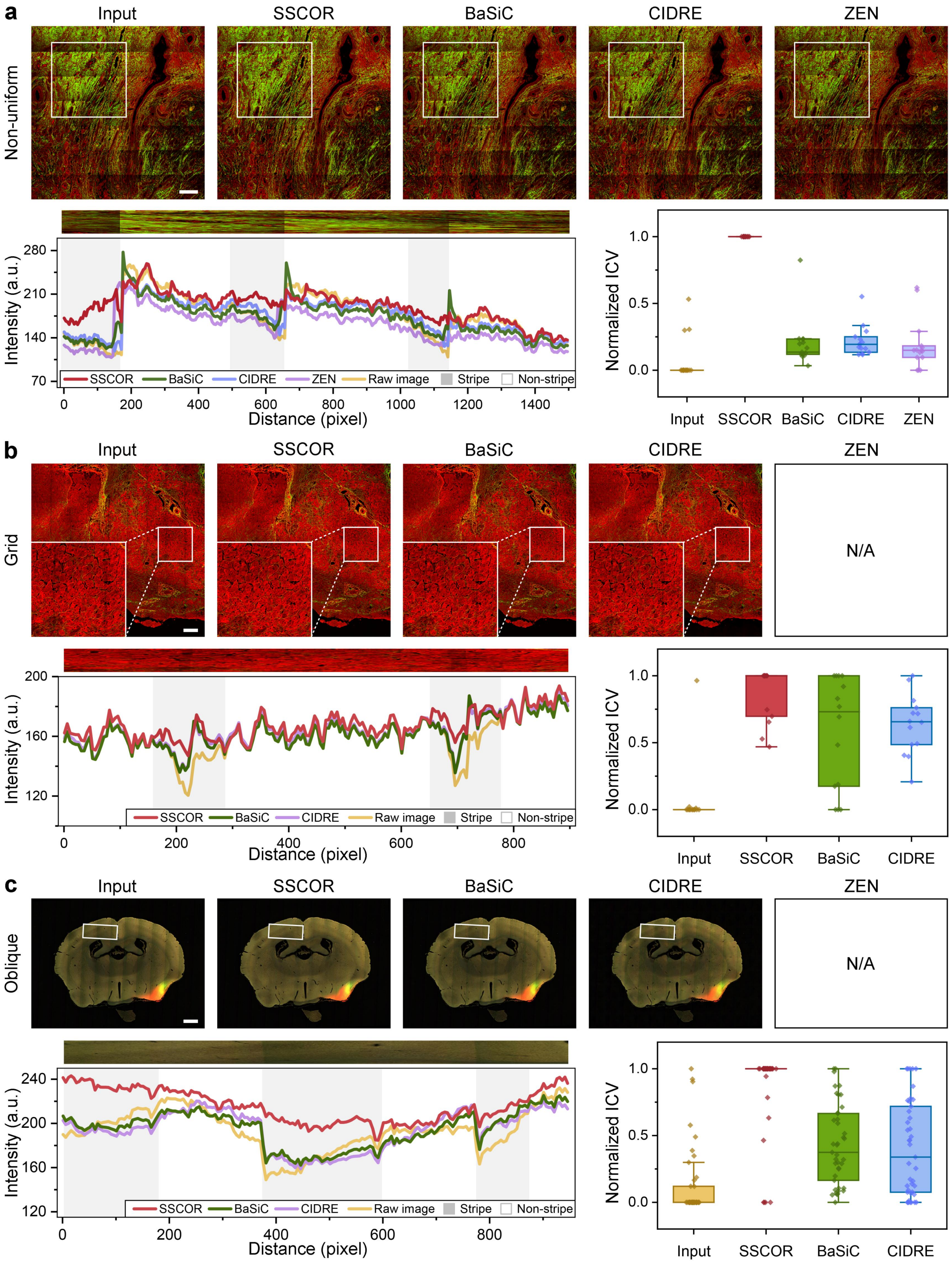

Supplementary Figure 2. Other representative stripe correction results. a), b), and c) Representative correction results and the corresponding quantitative analysis of non-uniform stripe (label-free multiphoton image of breast cancer), grid stripe (label-free multiphoton image of liver cancer), and oblique stripe (labeled fluorescence image of mouse brain[9]). SSCOR performs the state-of-the-art performance for stripe correction compared with the comparison approaches. The Zeiss correction method is not applicable (N/A) because there are no raw files for the grid and oblique stripe images. $n_{non\text{-}uniform} = 15$, $n_{grid} = 14$, $n_{oblique} = 41$. n represents the quantity of the stripe images. The size of the images varies depending on the stripe direction and the homogeneity of histopathological features. Box plots indicate median (middle line), 25th, 75th percentile (box) and 1.5 × interquartile range (whiskers). Source data are provided as a Source Data file. Scale bars in a) and b): 200 μm. Scale bars in c): 1 mm.

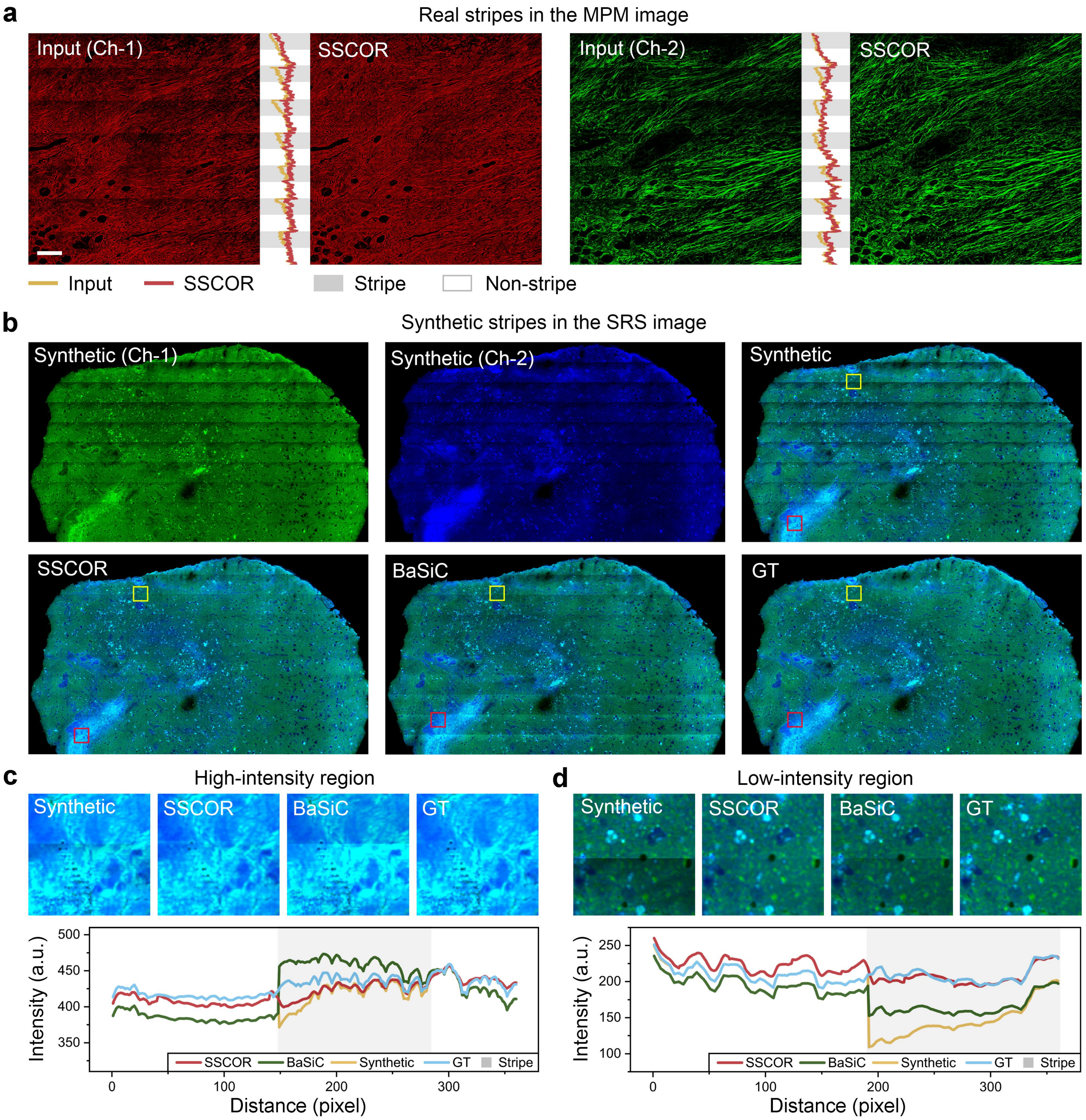

Supplementary Figure 3. Unbiased signal estimation across intensity levels of heterogeneous images. a) The intensity profiles of the stripe and non-stripe regions in the MPM image with real stripes. SSCOR is capable of correcting stripes and preserving the fluorescence intensity contrast in non-stripe regions in two heterogeneous channels. Scale bar: 200 μm. b) Comparison of correction effects in the heterogeneous SRS image with synthetic stripes. c) and d) The enlarged high-intensity region (red boxes) and low-intensity region (yellow boxes) in b). The intensity profiles demonstrated that SSCOR does not overestimate or underestimate fluorescence signal in stripe regions, while still preserving the intensity contrast of tissue components in non-stripe regions. Source data are provided as a Source Data file.

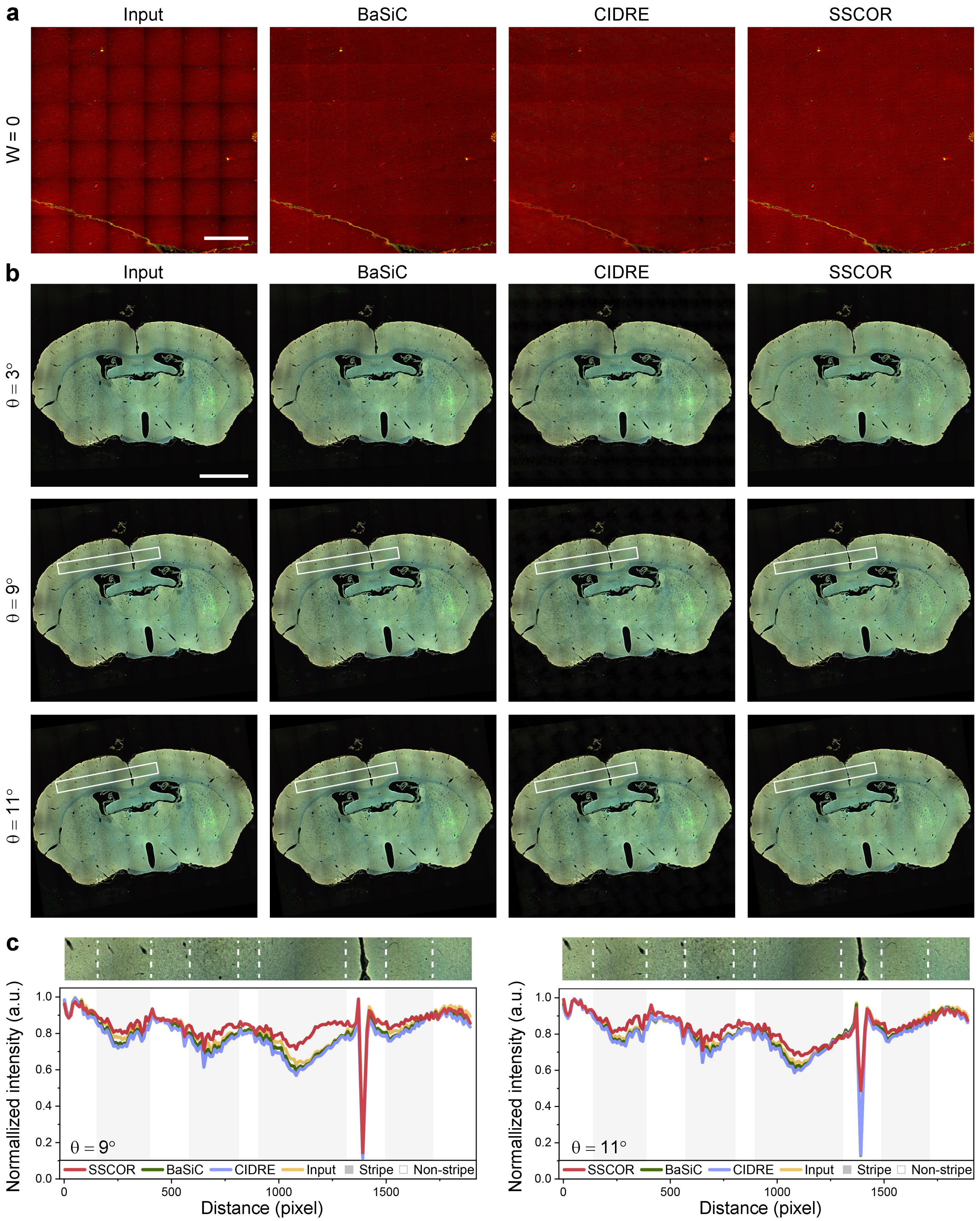

Supplementary Figure 4. Detailed correction results of imprecise prior stitched information. a) The comparative results of CIDRE[12] and BaSiC[11] under the ROI (region of interest) cropping conditions. Scale bar: 1 mm. b) The corrected results under the condition of greater image rotation. The intensity profiles within the rectangular regions quantitatively demonstrate that SSCOR has a better correction effect than other comparison methods when the angle of oblique stripes (θ) is no more than 11 degrees. Scale bar: 2 mm. c) The intensity profiles within the rectangular areas in b). Source data are provided as a Source Data file.

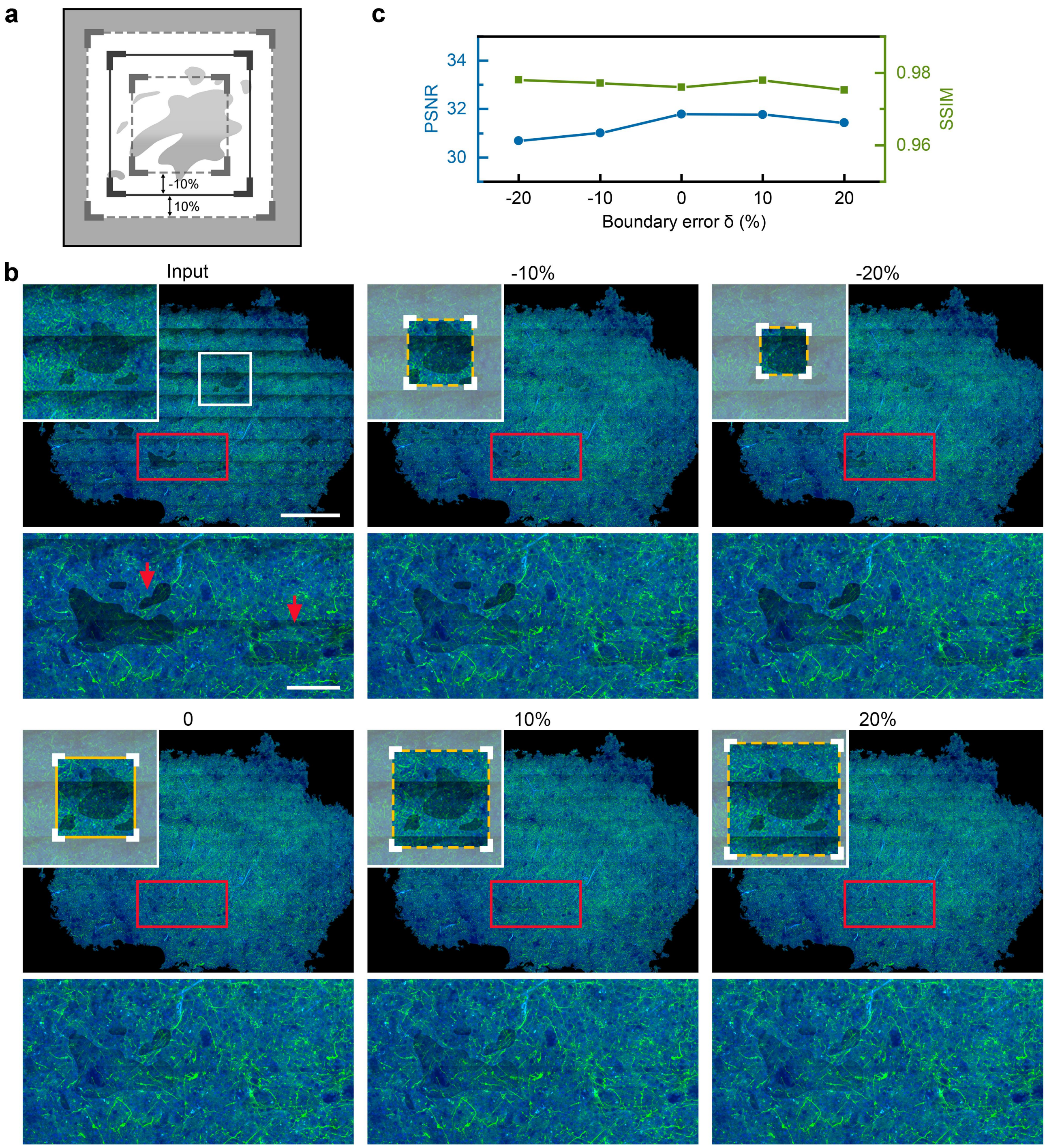

Supplementary Figure 5. SSCOR has tolerance towards the imprecise user-defined abnormal region for bubble-like artifacts. a) We illustrate the user-defined abnormal region for sampling anomaly patches from bubble-like artifacts. As shown in the schematic diagram, the user-defined boundary can be roughly adjusted to zoom in or out by 10%. b) and c) The yellow boxes represent the user-defined abnormal regions for bubble-like artifacts with different errors. We present the correction results obtained by sampling from different abnormal regions, with the enlarged and highlighted local regions within red boxes. Furthermore, we measure the corresponding peak signal-to-noise ratio (PSNR) and structural similarity index (SSIM). Our observations reveal that SSCOR effectively corrects artifacts, even when the boundary error ranges from -20% to 20%. Note that the superior artifact correction can be achieved by sampling anomaly patches from an abnormal region that is equal to or larger in size than the artifact itself. This suggests that the abnormal region can be slightly larger than the artifact to attain a high-quality correction outcome. Source data are provided as a Source Data file. Scale bar of input image: 1mm. Scale bar of enlarged image: 250 μm.

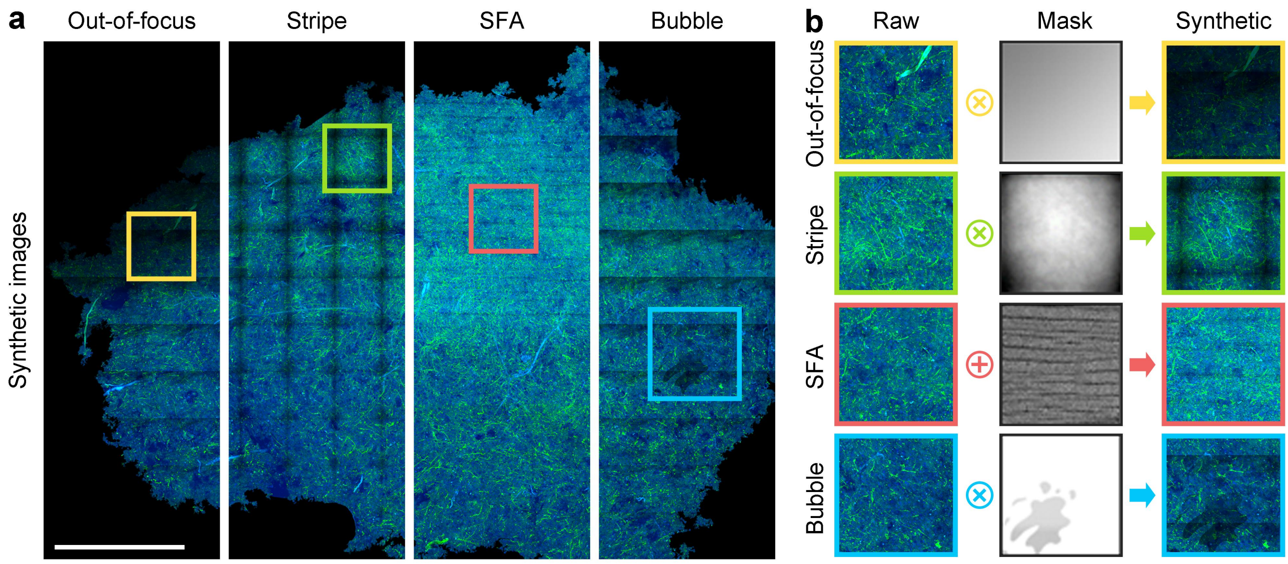

Supplementary Figure 6. Pipeline of the stripe and artifact synthesis. a) The synthetic images with non-uniform stripe, bubble-like artifacts, out-of-focus artifacts, and scanning fringe artifacts (SFA) based on SRS images[10]. b) The intensity attenuation of mask is added to specific regions to simulate the stripe and artifacts of the stitched image. Scale bar: 1 mm.

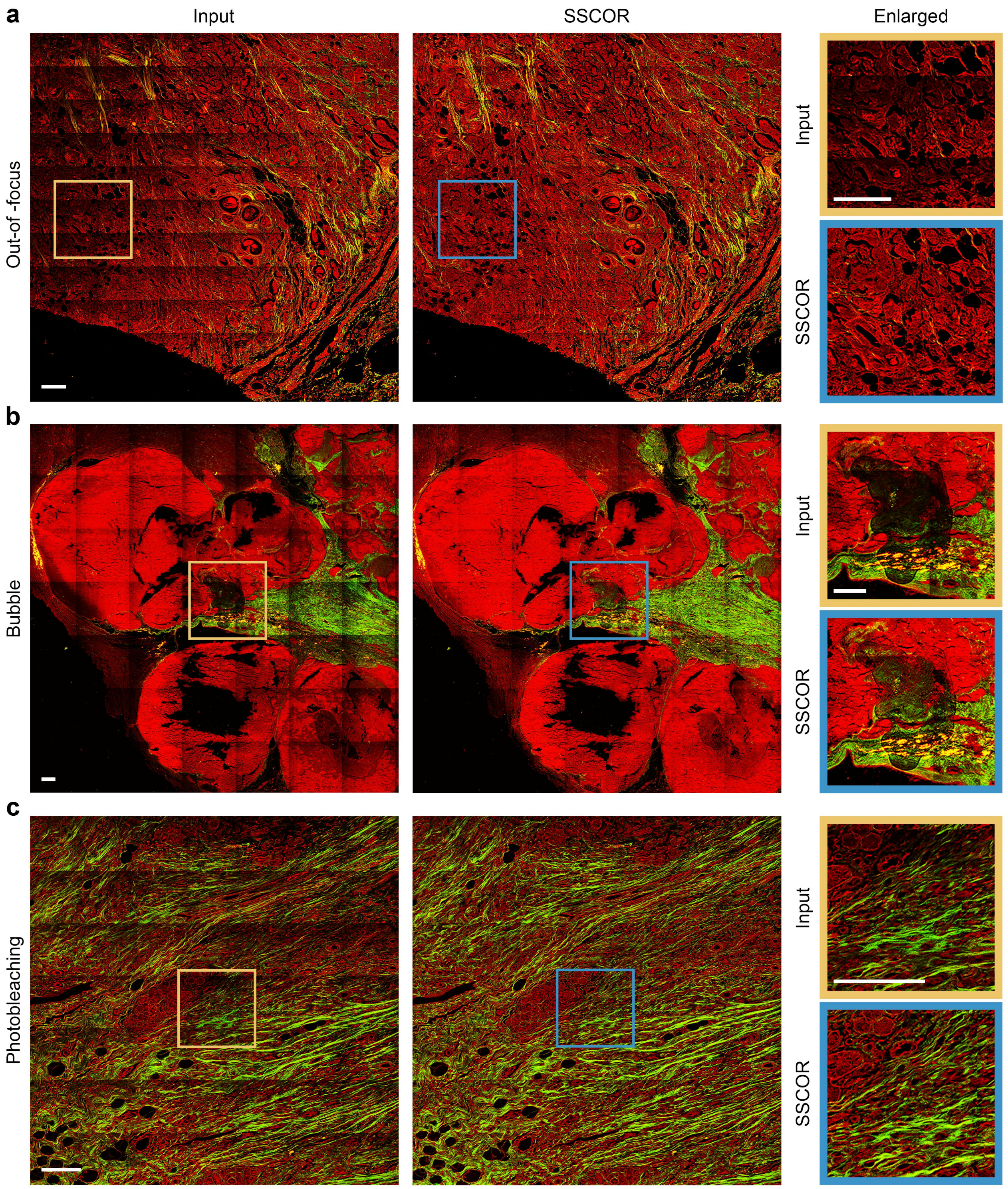

Supplementary Figure 7. Restoration results of real artifacts in the MPM images. a) Compared with the input image, SSCOR can restore out-of-focus areas in the real MPM image of breast cancer while removing stripes. b) SSCOR recovers the original tissue signal obscured by bubble-like artifacts (yellow box) in MPM images of cerebral cavernous malformations. c) In imaging experiments, repeated scanning on the same position is prone to photobleaching artifacts (yellow box). SSCOR restores the photobleaching signal and correct stripe in the MPM image of breast cancer. Scale bars: 200 μm.

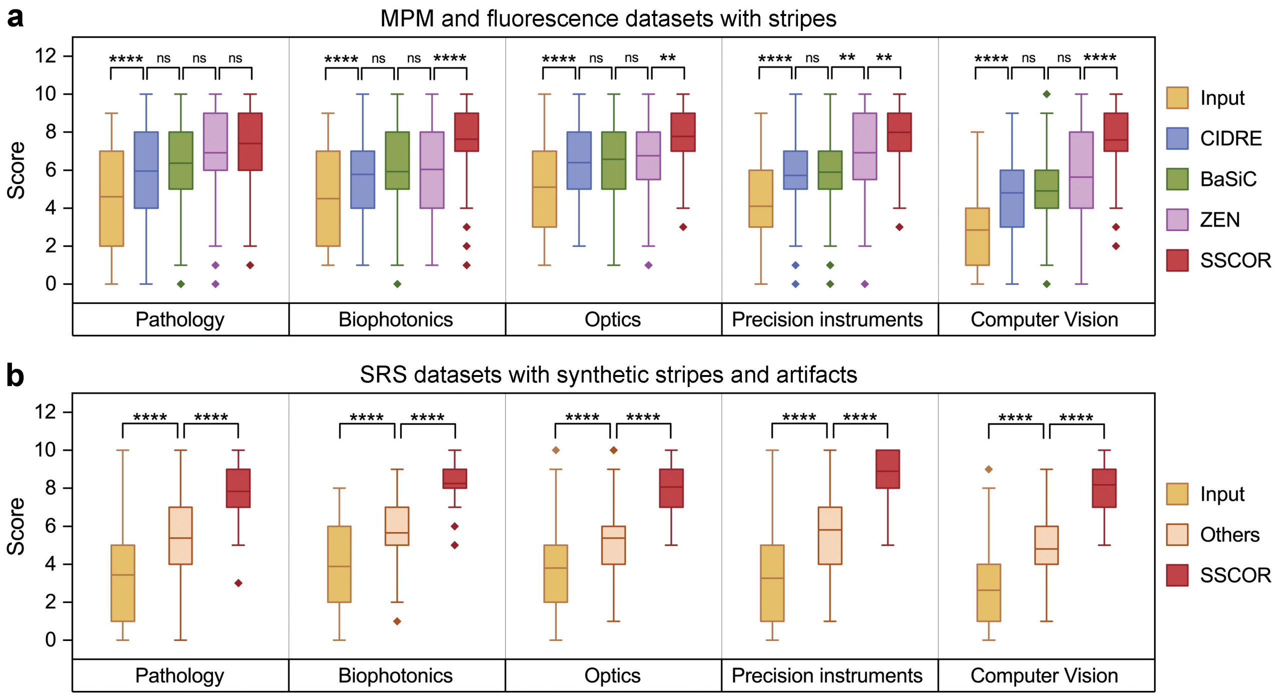

Supplementary Figure 8. User study results based on the background of participants. a) SSCOR achieved superior performance on MPM and fluorescence[9] datasets for different backgrounds (Nine participants in the pathology background are from the Affiliated Union Hospital and the First Affiliated Hospital of Fujian Medical University, respectively. Eleven participants in the biophotonics background are from Fujian Normal University. Ten participants in the optics background are from Xi'an Jiaotong University and Jilin University, respectively. Ten participants in the precision instruments background and ten participants in computer vision background are from Fuzhou University). n=702 images tested by pathology ($P_{Input\ vs.\ CIDRE} < 0.0001$, $P_{CIDRE\ vs.\ BaSiC} = 0.434$, $P_{BaSiC\ vs.\ ZEN} = 0.526$, $P_{ZEN\ vs.\ SSCOR} = 0.6182$). n=858 images tested by biophotonics ($P_{Input\ vs.\ CIDRE} < 0.0001$, $P_{CIDRE\ vs.\ BaSiC} = 0.9569$, $P_{BaSiC\ vs.\ ZEN} = 0.996$, $P_{ZEN\ vs.\ SSCOR} < 0.0001$). n=780 images tested by optics ($P_{Input\ vs.\ CIDRE} < 0.0001$, $P_{CIDRE\ vs.\ BaSiC} = 0.8824$, $P_{BaSiC\ vs.\ ZEN} = 0.9642$, $P_{ZEN\ vs.\ SSCOR} = 0.0015$). n=780 images tested by precision instruments ($P_{Input\ vs.\ CIDRE} < 0.0001$, $P_{CIDRE\ vs.\ BaSiC} = 0.9268$, $P_{BaSiC\ vs.\ ZEN} =0.0058$, $P_{ZEN\ vs.\ SSCOR} = 0.0034$). n=780 images tested by computer vision ($P_{Input\ vs.\ CIDRE} < 0.0001$, $P_{CIDRE\ vs.\ BaSiC} = 0.9911$, $P_{BaSiC\ vs.\ ZEN} = 0.0578$, $P_{ZEN\ vs.\ SSCOR} < 0.0001$). b) SSCOR outperformed other methods in correcting the SRS[10] datasets with synthetic stripes and artifacts. Other methods include ZeroDCE[2], BaSiC[11], N2N[7], and Mask-ShadowGAN[5]. n=216 images tested by pathology. n=264 images tested by biophotonics. n=240 images tested separately by optics, precision instruments, and computer vision. In the above backgrounds, all $P_{Input\ vs.\ Others} < 0.0001$, $P_{Others\ vs.\ SSCOR} < 0.0001$. Box plots indicate median (middle line), 25th, 75th percentile (box) and 1.5 × interquartile range (whiskers). One-way analysis of variance (ANOVA) followed by Tukey's multiple comparison test were used for a) and b). The significance level is displayed as asterisks, and $P < 0.05$ was considered statistically significant ($^{*}P < 0.05$, $^{**}P < 0.01$, $^{***}P < 0.001$, $^{****}P < 0.0001$; ns, not significant). Source data are provided as a Source Data file.

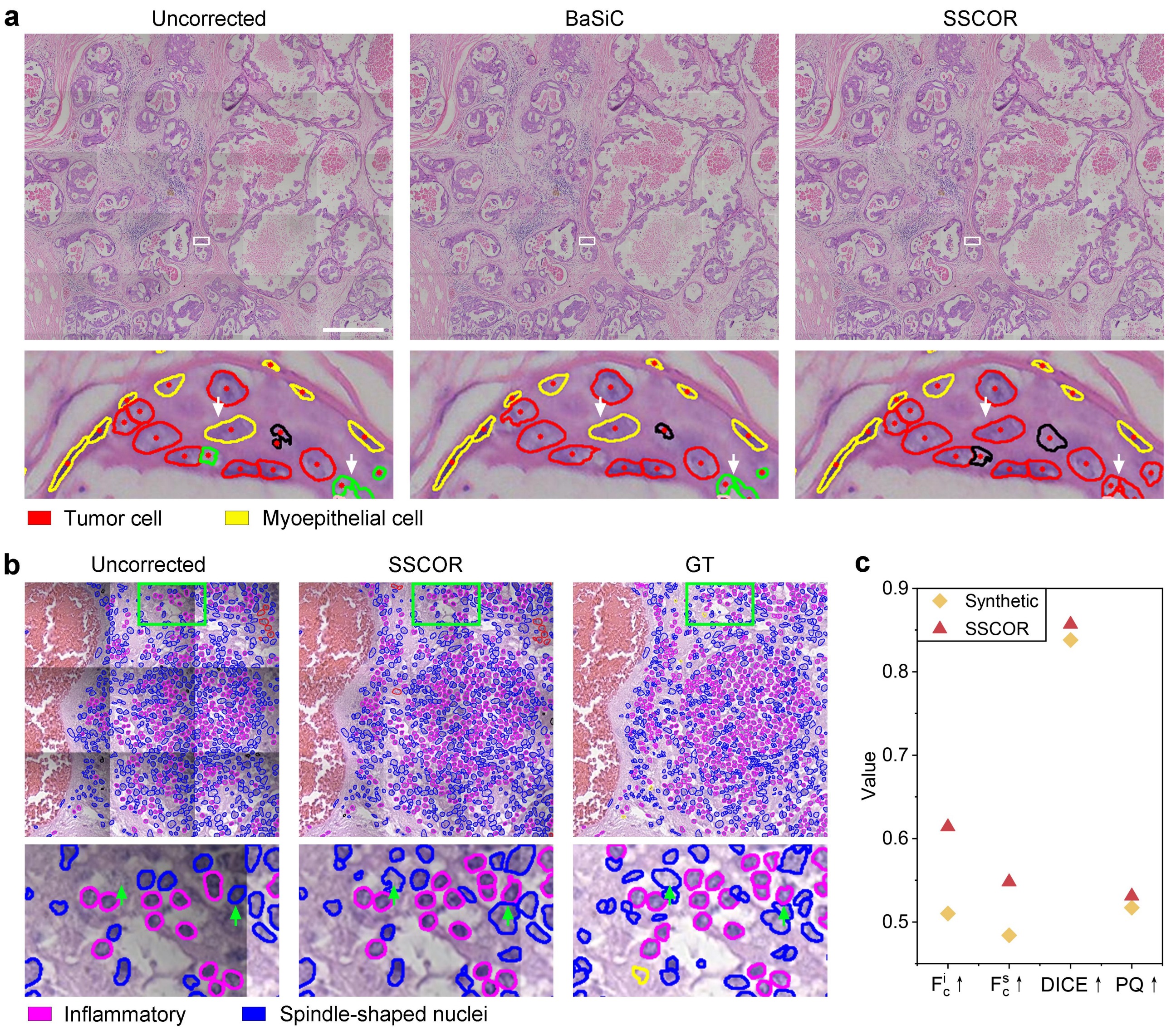

Supplementary Figure 9. Additional representative cell classification results based on SSCOR-corrected images. a) The complete correction results of the real H&E image in Fig. 5c, and the enlarged classification results of another stripe-free region (white box). Different colors of the nuclear boundaries denote separate instances. The white arrows point to the classified tumor cells, as confirmed by experienced pathologists. Scale bar: 1mm. b) The correction and classification results of a synthetic stripe from the H&E dataset, CoNSep. The green arrows indicate that the cell classification of SSCOR is consistent with ground-truth (GT). c) Four quantitative metrics further demonstrated that SSCOR-corrected images have the capability of improving segmentation and classification performance. F^i_c and F^s_c denote the F_1 classification score for the inflammatory and spindle-shaped nuclei classes respectively. Dice coefficient (DICE) measures the separation of nuclei from the background. Panoptic quality (PQ) represents a unified score for measuring the performance of nuclear instance segmentation methods. Source data are provided as a Source Data file.

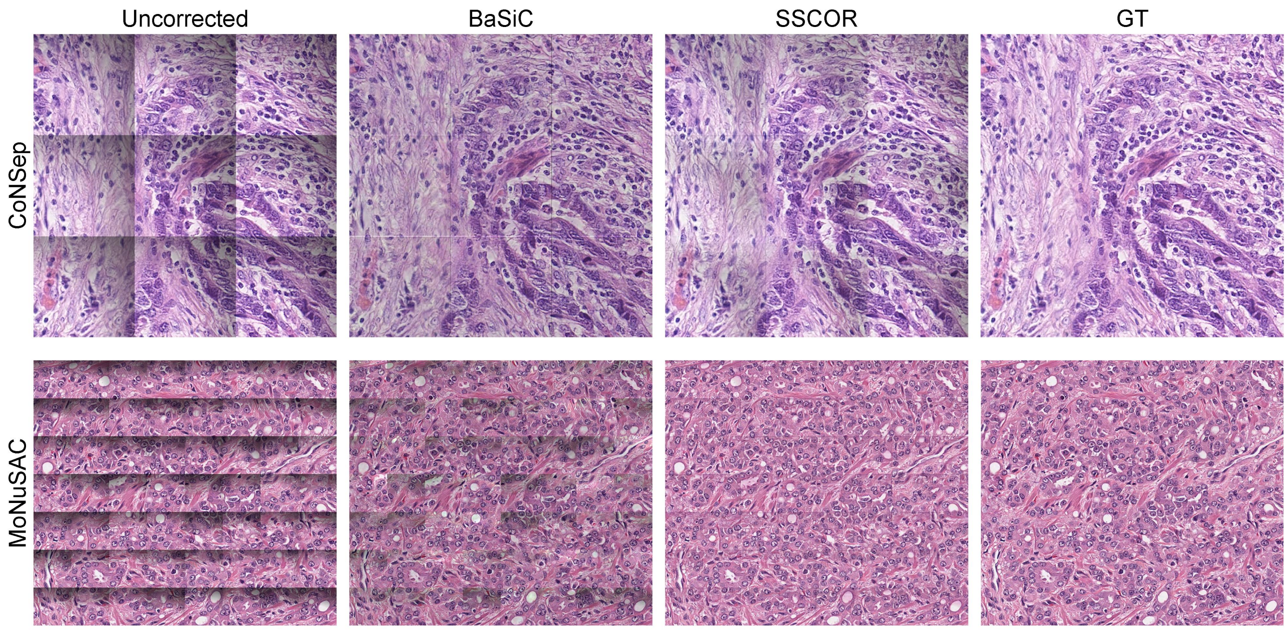

Supplementary Figure 10. Representative correction results of synthetic stripes on H&E images. We synthesized non-uniform stripes on the H&E images from two public datasets, CoNSep[14] and MoNuSAC[15]. Comparing to BaSiC, SSCOR obtains better stripe correction results. GT: ground truth.

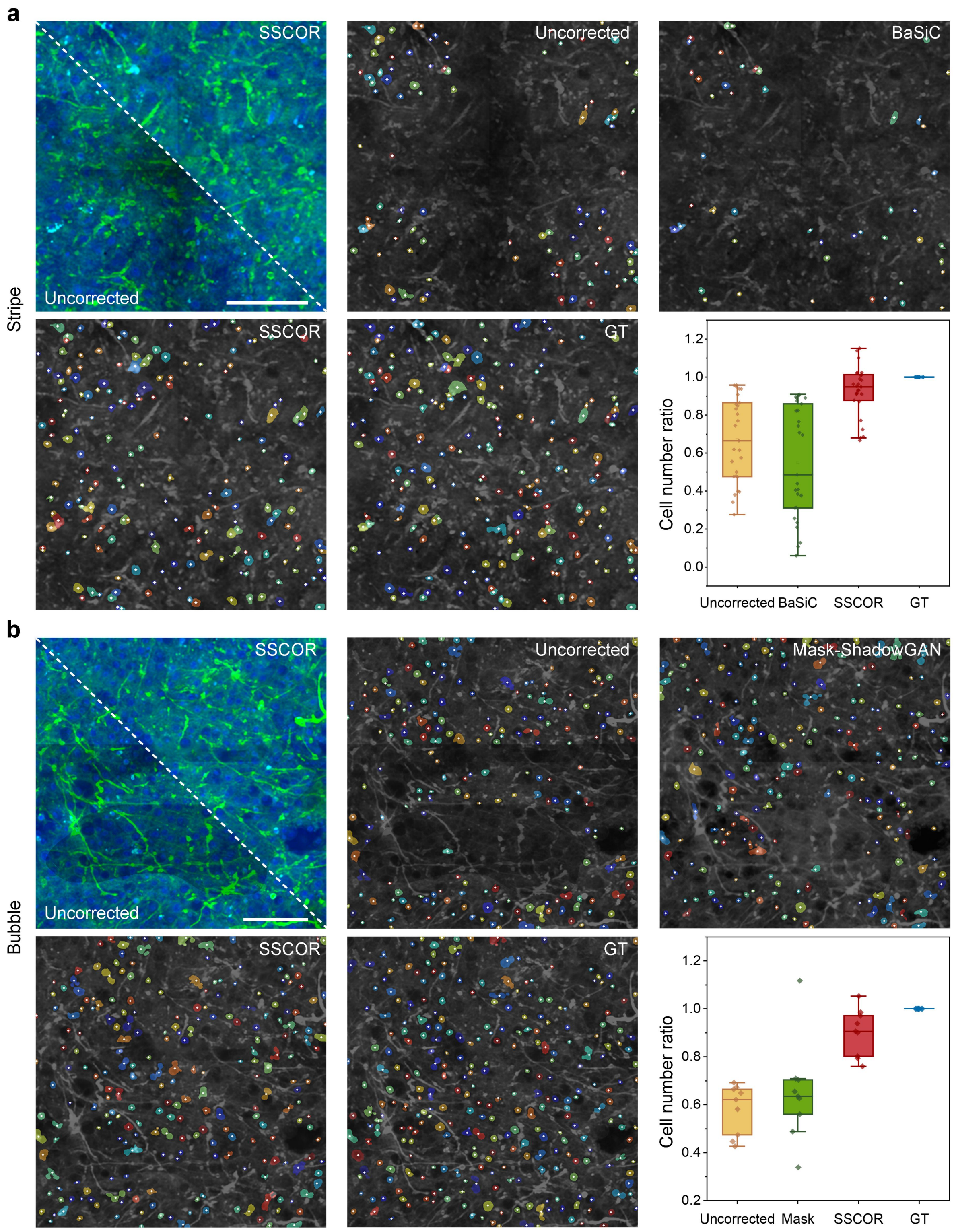

Supplementary Figure 11. Cell counting results on bubble artifact and stripe images. a) and b) Comparison of SSCOR-corrected images with uncorrected images, the automatic cell counting results of SSCOR are more consistent with ground-truth (GT) image. The original SRS image[10] before synthesis is considered as GT image. The white dots represent the location of cells, and the color-coded markers represent the size of cells. Scale bars: 100 μm. The cell number ratio quantitatively demonstrates the consistency of SSCOR-corrected and GT images ($n_{Stripe} = 27$ and $n_{Bubble} = 8$; n represents the quantity of ROIs in bubble-like artifact or stripe images). The cell number ratio is calculated by the ratio of cell numbers in the uncorrected/corrected image to the GT images. Box plots indicate median (middle line), 25th, 75th percentile (box) and 1.5 × interquartile range (whiskers). Source Data are provided as a Source Data file.

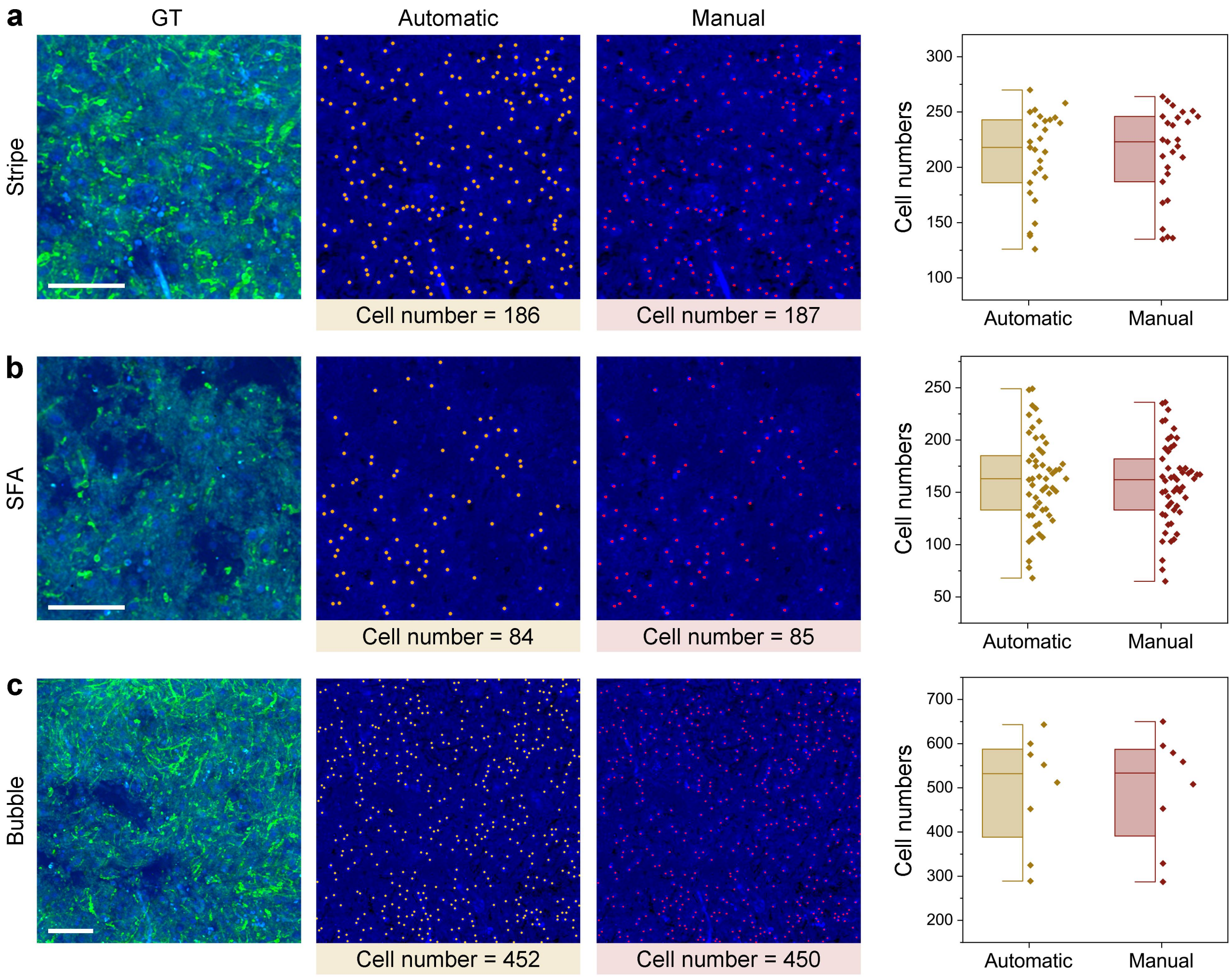

Supplementary Figure 12. Accuracy of automatic counting compared with manual counting. a), b), and c) The comparison examples of automatic and manual cell counting on scanning fringe artifacts (SFA), bubble artifacts, and stripe images. The automatic cell counting[16] is performed on blue channel of SRS images[10]. The dots indicate the location of the cells. Scale bars: 100 μm. The quantitative results of cell numbers validate the accuracy of automatic counting ($n_{Stripe} = 27$, $n_{SFA} = 53$, $n_{Bubble} = 8$; n represents the quantity of ROIs in artifacts or stripe images). Box plots indicate median (middle line), 25th, 75th percentile (box) and 1.5 × interquartile range (whiskers). Source data are provided as a Source Data file.

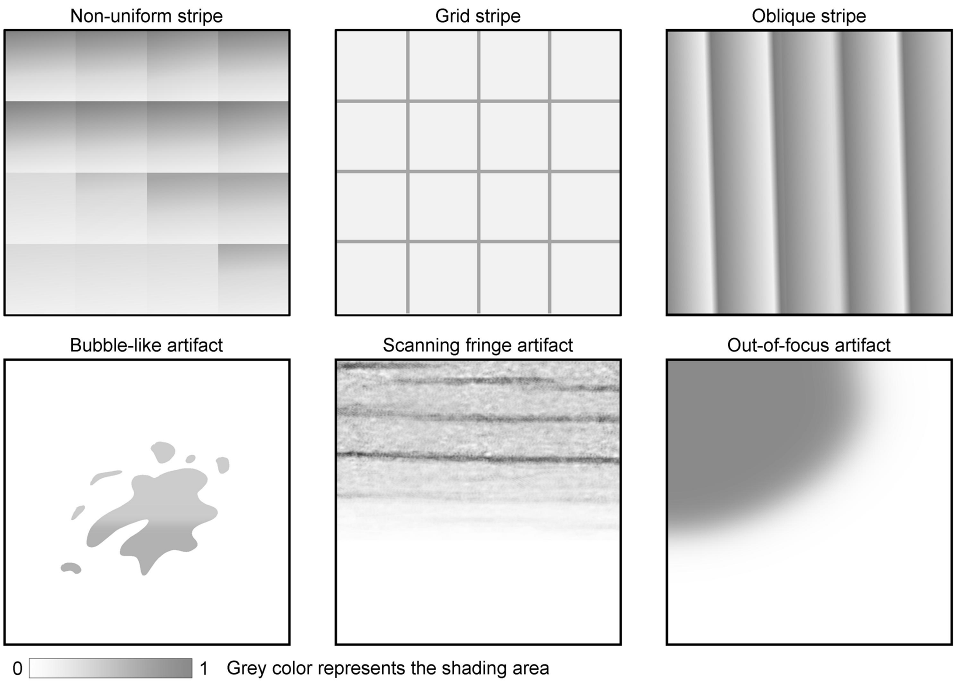

Supplementary Figure 13. **Schematic diagram of typical stripes and artifacts.** We show the diagrams of non-uniform stripe, grid stripe, oblique stripe, bubble-like artifact, scanning fringe artifact, and out-of-focus artifact. The sampling strategy of the stripes and artifacts are outlined in Supplementary Table 4.

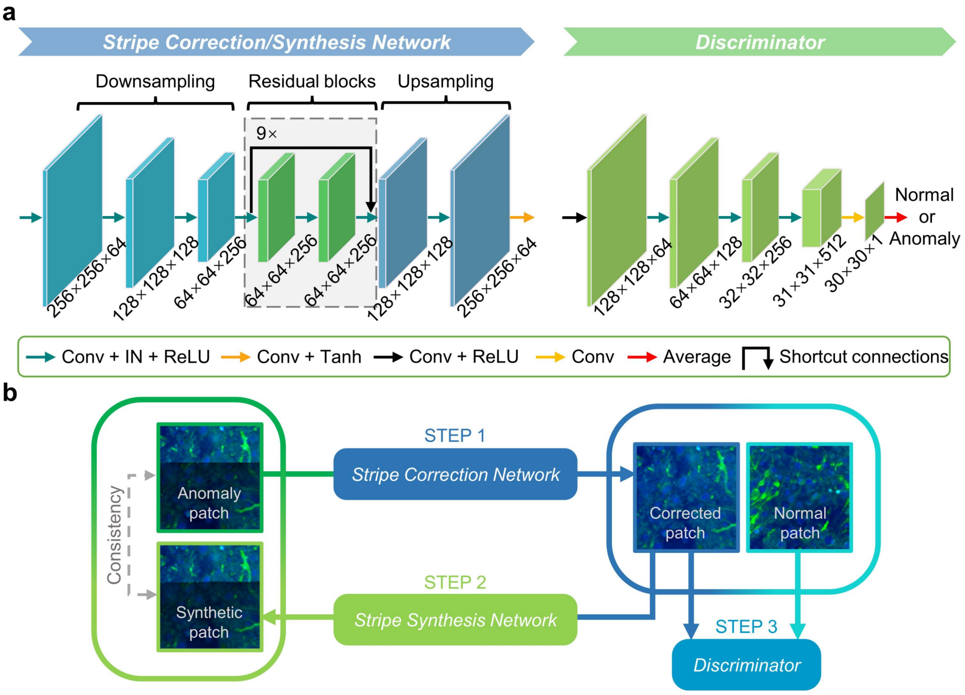

Supplementary Figure 14. Visualization of network architecture. a) The stripe correction network consists of downsampling layers, intermediate layers, and upsampling layers. The discriminator network consists of five convolutional layers. b) The diagram shows the image data flows during the adversarial self-training stage of SSCOR. Each cube in a) represents a multi-channel feature map, and the label below each cube indicates the spatial dimensions and channel information specific to that feature map. Conv, convolution layers; IN, Instance Normalization; ReLU, Rectified Linear Unit; Tanh, Hyperbolic Tangent; Shortcut connections, the information flow skipping one or more layers; 9 ×, the cascading nine identical residual blocks.

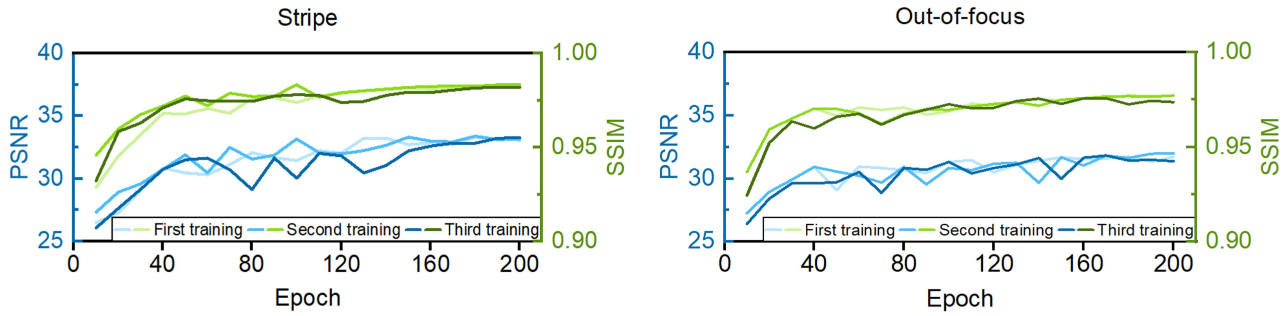

Supplementary Figure 15. Model performance versus the training epochs with random initialization. We show the training curves with regards to the correction quality for the synthesized stripes and out-of-focus artifacts (Fig. 4a), respectively. For each synthesized image, model training is performed for three times with random initialization, which was measured in PSNR and SSIM for every 10 epochs until it reaches 200 epochs. As observed, both PSNR and SSIM exhibit an increasing trend with more training epochs. Although three training curves show fluctuations during training process, they eventually converge to similar results at the 200th epoch.

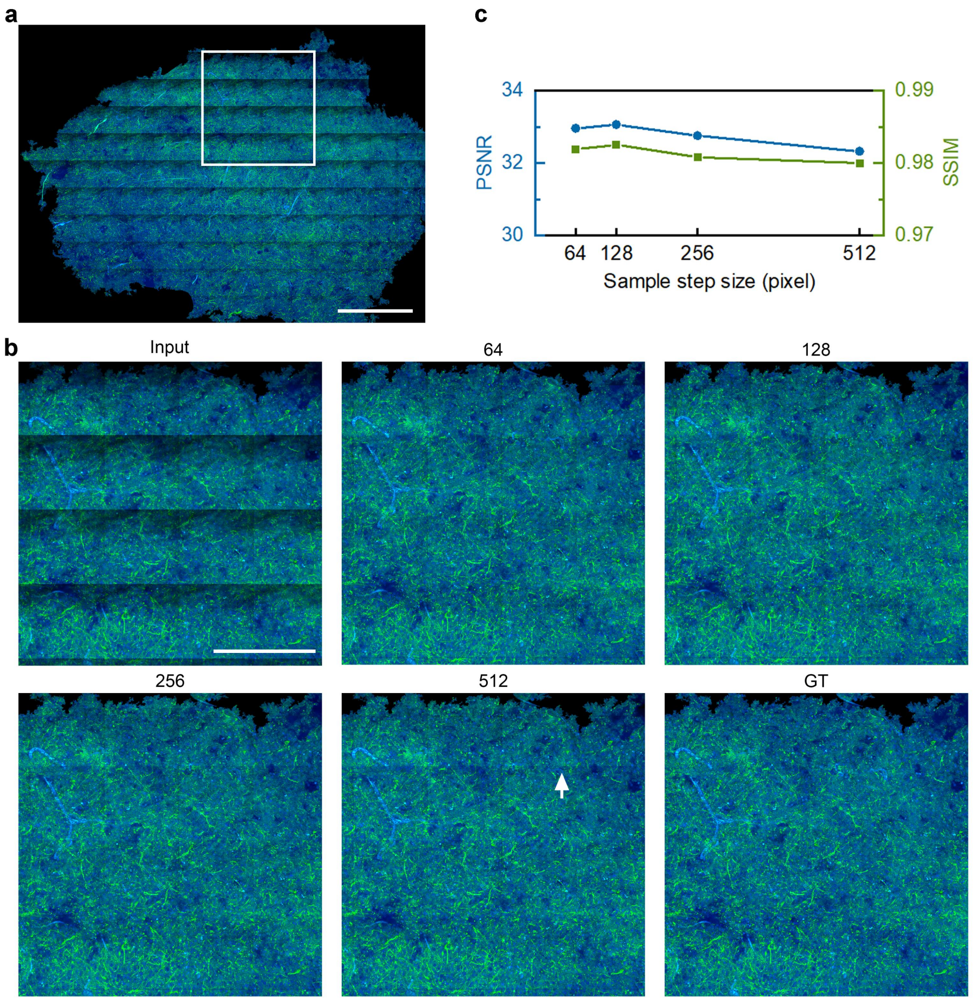

Supplementary Figure 16. The effect of sampling step size on model performance. a) and b) We adopted different step sizes during training, and SSCOR showed the stable performance with the step sizes of 64, 128, or 256. Despite a slight performance drop at the step size of 512, SSCOR is able to well correct stripes. The white arrow indicates the partially uncorrected stripes when the step size is 512. Scale bar of a): 1 mm. Scale bar of b): 500 µm. c) The PSNR and SSIM curves for the corrected images exhibit minimal fluctuations, indicating the stability of SSCOR.

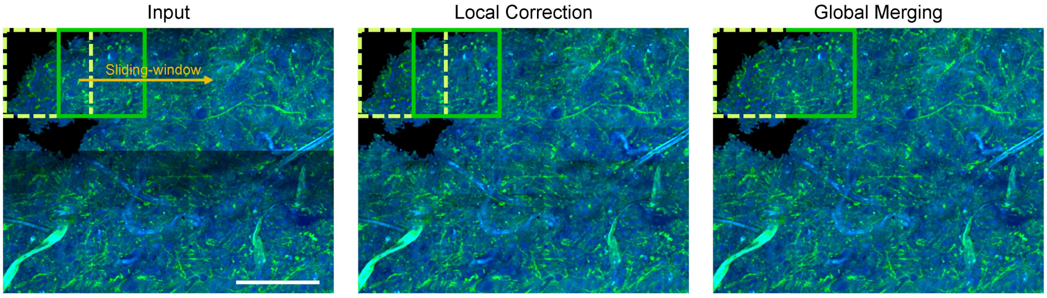

Supplementary Figure 17. Illustration of the local-to-global strategy. The local-to-global strategy consists of the local correction and global merging steps. The local correction step involves partitioning the input stitched image into overlapping local patches using a sliding-window approach. Each local patch is then processed by the well-trained stripe correction network. In the global merging step, the corrected patches are merged and the overlapped region of adjacent local corrected patches are averaged to reconstruct the complete image. In practice, the sliding-window step size is set to be smaller than the patch size, to ensure that adjacent patches overlap sufficiently to enable the smoothness of merging. Scale bar: 200 µm.

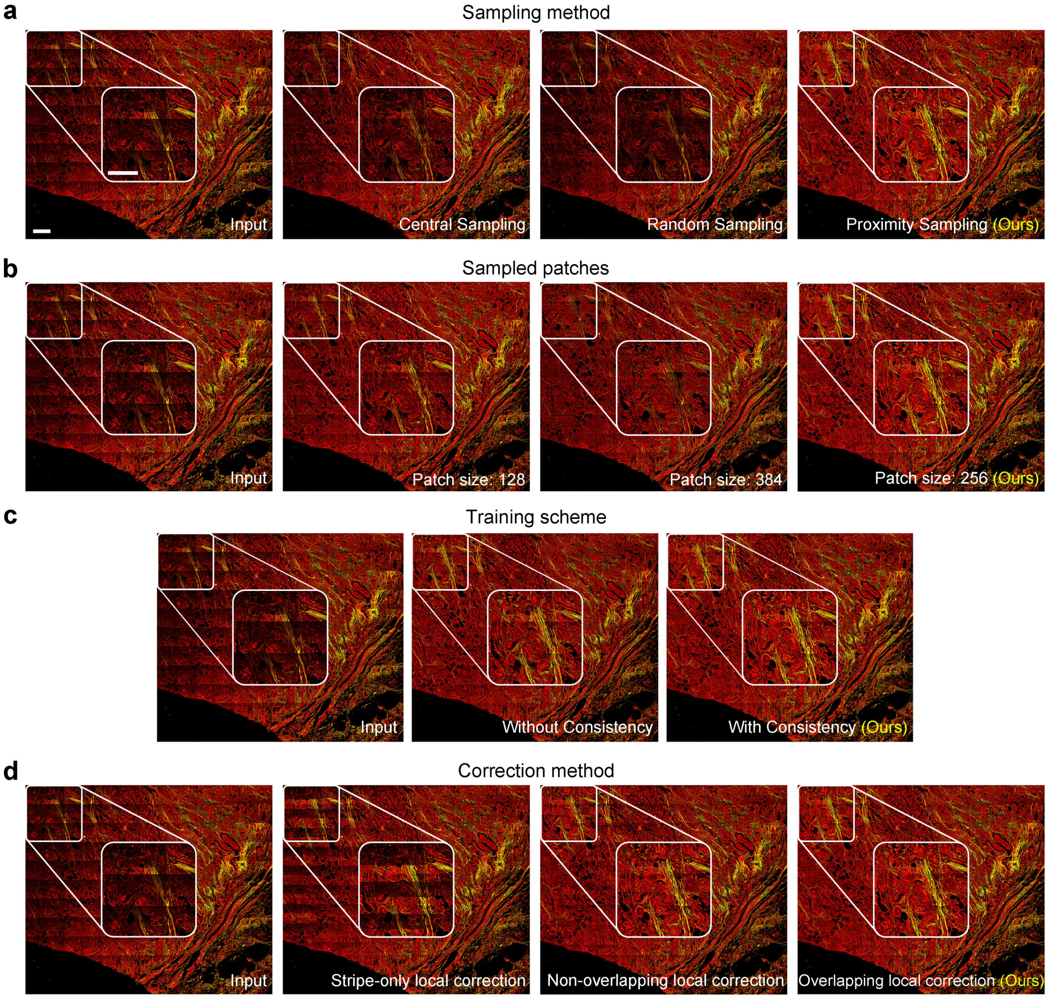

Supplementary Figure 18. Ablation study. a) Ablation study on the sampling method by comparing the correction results of the proposed proximity sampling against central sampling and random sampling. b) Ablation study on the size of sampled patches by setting different patch sizes for quantitative comparison. c) Ablation study on the training scheme. The experiments are performed to assess the importance of consistency constrain for stripe correction. d) Ablation study on different ways of merging the local correction results into the final result. Scale bar: 250 µm.

Supplementary Tables

Supplementary Table 1. User study results of MPM and fluorescence[9] datasets with stripes. Fifty participants from diverse backgrounds were asked to rate the test images on a severity scale of 0 to 10 according to the presence of stripes and artifacts. Higher scores indicate better stripe correction. Results are presented as mean±SEM. Not applicable (N/A) denotes the absence of the corresponding data. Source data are provided as a Source Data file.

<table>
<tr><th></th><th>Input</th><th>BaSiC[11]</th><th>CIDRE[12]</th><th>ZEN[13]</th><th>SSCOR</th></tr>
<tr><td>Non-uniform</td><td>3.73 ± 2.31</td><td>5.28 ± 2.09</td><td>5.21 ± 1.98</td><td>6.43 ± 2.52</td><td>7.93 ± 1.60</td></tr>
<tr><td>Grid</td><td>3.97 ± 2.24</td><td>6.52 ± 1.95</td><td>6.30 ± 1.80</td><td rowspan="2">N/A</td><td>6.99 ± 1.80</td></tr>
<tr><td>Oblique</td><td>4.98 ± 2.17</td><td>5.96 ± 1.90</td><td>5.67 ± 1.90</td><td>8.11 ± 1.41</td></tr>
<tr><td>Total</td><td>4.23 ± 2.31</td><td>5.92 ± 2.04</td><td>5.73 ± 1.95</td><td>6.43 ± 2.52</td><td>7.68 ± 1.68</td></tr>
</table>

Supplementary Table 2. User study results of SRS[10] datasets with synthetic stripes and artifacts. The comparison methods include ZeroDCE[2], BaSiC[11], N2N[7], and Mask-ShadowGAN[5]. Results are presented as mean±SEM. Higher scores mean better stripe correction. Source data are provided as a Source Data file.

	Input	Other methods[2,5,7,11]	SSCOR
**Out-of-focus**	2.88 ± 2.21	4.89 ± 1.77	8.57 ± 1.15
**Stripe**	2.62 ± 2.13	5.57 ± 1.81	8.25 ± 1.34
**SFA**	5.58 ± 2.25	5.53 ± 2.20	8.28 ± 1.24
**Bubble**	2.57 ± 2.16	5.64 ± 1.97	7.91 ± 1.24
Total	3.41 ± 2.52	5.41 ± 1.96	8.25 ± 1.26

Supplementary Table 3. Requirements of different methods for training images.

<table>
<tr><th rowspan="3">Method</th><th colspan="5">Requirements for Image Training</th></tr>
<tr><th colspan="2">a 6348×5376 pixels stitched image with 13×11 tiles</th><th colspan="2">a 2881×2872 pixels stitched image with 3×3 tiles</th><th rowspan="2">Image Format</th></tr>
<tr><th>Sample Number</th><th>Sample Size (pixel)</th><th>Sample Number</th><th>Sample Size (pixel)</th></tr>
<tr><td>ZEN[13]</td><td rowspan="3">143 tiles</td><td rowspan="3">512×512</td><td rowspan="3">9 tiles</td><td rowspan="3">1024×1024</td><td>.czi</td></tr>
<tr><td>BaSiC[11]</td><td rowspan="2">.tif</td></tr>
<tr><td>CIDRE[12]</td></tr>
<tr><td>Neighbor2Neighbor[7]</td><td rowspan="2">633 patches</td><td rowspan="2">256×256</td><td rowspan="2">166 patches</td><td rowspan="2">256×256</td><td rowspan="2">.tif</td></tr>
<tr><td>ZeroDCE[2]</td></tr>
<tr><td>Mask-ShadowGAN[5]</td><td rowspan="2">633 unpaired patches</td><td rowspan="2">256×256</td><td rowspan="2">166 unpaired patches</td><td rowspan="2">256×256</td><td rowspan="2">.tif</td></tr>
<tr><td>SSCOR</td></tr>
</table>

Supplementary Table 4. Description of stripe and artifact types and the corresponding sampling strategy.

<table>
<tr><th>Type</th><th>Description</th><th>Causes</th><th>Sampling strategy</th></tr>
<tr><td>Non-uniform stripe</td><td>The stitched image presents non-uniform stripe pattern, in which each tile exhibits diverse shading patterns.</td><td>Multiple factors like the condition of specimen or microscope result in non-uniformity.</td><td rowspan="3">Since the exact positions of stripes in stitched images are unknown, we assume that the stripes are uniformly distributed and perpendicular to the image boundaries. This assumption applies even to images with oblique stripes. The sampling steps are as follows:
1. Estimate the position of each stripe in the stitched image based on image width, height, and the number of stripes.
2. Sample anomaly patches along each estimated horizontal or vertical stripe using a sliding window approach.
3. The striped area covered by the sampling is considered the abnormal region, with the remaining non-striped area being the normal region.
4. Once an anomaly patch is sampled, the corresponding normal patch can be obtained from the nearby non-striped areas using the proposed strategy (please refer to Proximity Sampling Strategy in Methods).</td></tr>
<tr><td>Grid stripe</td><td>The stitched image presents horizontal and vertical stripes that resemble a grid pattern, in which each tile shares the same shading pattern.</td><td>The shading of each tile is caused by uneven illumination.</td></tr>
<tr><td>Oblique stripe</td><td>The vertical or horizontal stripes of stitched images present slightly oblique.</td><td>Pathological tissues are commonly adhered askew on glass slide for microscopic examination, which may necessitate proper rotation of the acquired image to meet downstream application requirement. This rotation can lead to the presence of oblique stripes in the image.</td></tr>
<tr><td>Bubble-like artifact</td><td>The artifact appears bubble-like irregular shape with uneven shading.</td><td>The artifact is essentially local tissue moisture loss caused by laser photodamage or the unsealed specimen.</td><td rowspan="3">1. Localize the abnormal region through user interaction, where the user can draw a coarse region around the artifacts to define the abnormal region. The remaining image region can be considered as normal region.
2. Sample anomaly patches within the abnormal region, while normal patches can be sampled from adjacent normal regions outside the abnormal region.</td></tr>
<tr><td>Scanning fringe artifact</td><td>The artifact typically appears throughout the entire image, accompanied by a large area of noise.</td><td>The artifact is often produced by a high-speed galvo-resonant scanning imaging system.</td></tr>
<tr><td>Out-of-focus artifact</td><td>The artifact usually locates in the corner of the stitched image, which appears significantly reduction of the signal intensity within shading.</td><td>Due to the unevenness of the boundary area of the tissue sample, the microscope is out of focus on these areas.</td></tr>
<tr><td>Co-existed artifacts and stripes</td><td>The aforementioned artifacts overlap with the stripes in the stitched image.</td><td>Refer to the above reasons with regards to the type of stripe and artifact.</td><td>1. Apply the aforementioned strategies jointly to define abnormal regions encompassing both stripes and artifacts.
2. Sample anomaly patches from abnormal regions, while normal patches are sampled from nearby normal regions.</td></tr>
</table>

Supplementary Table 5. Comparison of the microscopic datasets.

Stripes/Artifacts types		Dataset	Specimen	Number	Resolution (pixel)	Microscope	Detector /Sensor	Objective	Immersion medium	Light Source	
										Type	Detail
Stripes	Non-uniform	H&E[14,15]	Colorectal adenocarcinoma	14	1000×1000	Omnyx VL120	-	40×	-	LED	-
			Breast cancer, kidney cancer, lung cancer, and prostate cancer	3	1600×1400<br>1800×1400<br>2000×1400	-	-	40×	-	LED	-
			Breast cancer	1	11143 ×9249	Motic VM1000	CCD	40×<br>0.95 NA	Water	LED	Electronically dimmable 6V/10W
		MPM	Breast cancer	10	3430×3430,	Zeiss LSM 880	PMT	20×<br>0.8 NA	Air	Coherent Laser	Chameleon Ultra<br>Ti: Sapphire<br>Femtosecond<br>Excitation wavelength (810 nm)<br>Average laser power (30 mW)
			Cerebral vascular malformation	1	5550×5550,						
	Grid		Liver cancer	4	4403×4403,						
	Oblique	Fluorescence[9]	Mouse brain	5	4171×3736,<br>4346×3080,	Hamamatsu NanoZoomer	TDI	20×<br>0.75 NA	Air	Hamamatsu Mercury lamp	LX2000<br>Ultrahigh-pressure<br>200 W
Artifacts	Out-of-focus	SRS[10]	Glioblastoma	2	7350×5390,	Olympus FV300	CCD	60×<br>1.2 NA	Water	APE GmbH Laser	picoEmerald<br>Tunable Two-Color Source<br>Picosecond<br>Optimal Raman shifts (2973, 2921, and 2851 cm^{-1})
	Bubble				2838×3892,						
	SFA										

H&E, Haemotoxylin and Eosin; MPM, multiphoton microscopy; PMT, photomultiplier; NA, numerical aperture; TDI, time delay integration; SRS, Stimulated Raman scattering; CCD, charge coupled device.

Supplementary Table 6. Proximity sampling on representative cases.

<table>
<tr><th colspan="2" rowspan="2">Stripes/Artifacts types</th><th rowspan="2">Specimen</th><th rowspan="2">Resolution(pixel)</th><th rowspan="2">Reference</th><th rowspan="2">Tiles</th><th colspan="3">Sample strategy</th></tr>
<tr><th>Step size (pixel)</th><th>Patch size (pixel)</th><th>Sample number</th></tr>
<tr><td rowspan="9">Stripes</td><td rowspan="5">Non-uniform</td><td rowspan="2">Cerebral vascular malformation</td><td>2881×2872</td><td>Supplementary Table 3</td><td>3×3</td><td>64</td><td rowspan="2">256×256</td><td>166</td></tr>
<tr><td>6387×6387</td><td>Supplementary Fig. 7b</td><td>7×7</td><td>128</td><td>576</td></tr>
<tr><td rowspan="3">Breast cancer</td><td>3430×3430</td><td>Supplementary Fig. 2a</td><td>7×7</td><td>128</td><td rowspan="2">256×256</td><td>198</td></tr>
<tr><td>6348×5376</td><td>Fig. 2a</td><td>11×13</td><td>128</td><td>633</td></tr>
<tr><td>11109×9122</td><td>Fig. 5c</td><td>5×5</td><td>256</td><td>512×512</td><td>316</td></tr>
<tr><td rowspan="3">Oblique</td><td rowspan="3">Mouse brain</td><td>4172×3737</td><td>Fig. 3b</td><td>9×10</td><td>64</td><td rowspan="3">256×256</td><td>210</td></tr>
<tr><td>4354×3086</td><td>Fig. 2a</td><td>8× 11</td><td>64</td><td>220</td></tr>
<tr><td>5000× 3750</td><td>Supplementary Fig. 2c</td><td>11×13</td><td>128</td><td>231</td></tr>
<tr><td>Grid</td><td>Liver cancer</td><td>4403×4403</td><td>Fig. 2a</td><td>9×9</td><td>128</td><td>128×128</td><td>544</td></tr>
<tr><td rowspan="3">Artifacts</td><td>Out-of-focus</td><td rowspan="3">Glioblastoma</td><td>7350×5390</td><td>Fig. 4</td><td>11×15</td><td>128</td><td rowspan="3">256×256</td><td>735</td></tr>
<tr><td>Bubble</td><td>7350×5390</td><td>Fig. 4</td><td>11×15</td><td>128</td><td>735</td></tr>
<tr><td>SFA</td><td>7350×5390</td><td>Fig. 4</td><td>11×15</td><td>128</td><td>470</td></tr>
</table>

Supplementary References

1 Shen, B. *et al.* Deep learning autofluorescence-harmonic microscopy. *Light Sci Appl* **11**, 76 (2022).

2 Guo, C. *et al.* Zero-reference deep curve estimation for low-light image enhancement. in *2020 IEEE/CVF Conference on Computer Vision and Pattern Recognition (CVPR)* 1780-1789 (2020).

3 Liu, R., Ma, L., Zhang, J., Fan, X. & Luo, Z. Retinex-inspired unrolling with cooperative prior architecture search for low-light image enhancement. in *2021 IEEE/CVF Conference on Computer Vision and Pattern Recognition (CVPR)* 10561-10570 (2021).

4 Ma, L., Ma, T., Liu, R., Fan, X. & Luo, Z. Toward fast, flexible, and robust low-light image enhancement. in *2022 IEEE/CVF Conference on Computer Vision and Pattern Recognition (CVPR)* 5637-5646 (2022).

5 Hu, X., Jiang, Y., Fu, C.-W. & Heng, P.-A. Mask-shadowgan: Learning to remove shadows from unpaired data. in *2019 IEEE/CVF International Conference on Computer Vision (ICCV)* 2472-2481 (2019).

6 Liu, Z., Yin, H., Mi, Y., Pu, M. & Wang, S. Shadow removal by a lightness-guided network with training on unpaired data. *IEEE Transactions on Image Processing* **30**, 1853-1865 (2021).

7 Huang, T., Li, S., Jia, X., Lu, H. & Liu, J. Neighbor2neighbor: Self-supervised denoising from single noisy images. in *2021 IEEE/CVF conference on computer vision and pattern recognition (CVPR)* 14781-14790 (2021).

8 Quan, Y., Chen, M., Pang, T. & Ji, H. Self2self with dropout: Learning self-supervised denoising from single image. in *2020 IEEE/CVF conference on computer vision and pattern recognition (CVPR)* 1890-1898 (2020).

9 Bohland, J. W. *et al.* A proposal for a coordinated effort for the determination of brainwide neuroanatomical connectivity in model organisms at a mesoscopic scale. *PLoS Comput Biol* **5**, e1000334 (2009).

10 Lu, F. K. *et al.* Label-Free Neurosurgical Pathology with Stimulated Raman Imaging. *Cancer Res* **76**, 3451-3462 (2016).

11 Peng, T. *et al.* A BaSiC tool for background and shading correction of optical microscopy images. *Nat Commun* **8**, 14836 (2017).

12 Smith, K. *et al.* CIDRE: an illumination-correction method for optical microscopy. *Nat Methods* **12**, 404-406 (2015).

13 ZEN Microscopy Software. https://www.zeiss.com/microscopy/en/products/software/zeiss-zen.html (2022).

14 Graham, S. *et al.* Hover-net: Simultaneous segmentation and classification of nuclei in multi-tissue histology images. *Medical Image Analysis* **58**, 101563 (2019).

15 Verma, R. *et al.* MoNuSAC2020: A multi-organ nuclei segmentation and classification challenge. *IEEE Transactions on Medical Imaging* **40**, 3413-3423 (2021).

16 Carpenter, A. E. *et al.* CellProfiler: image analysis software for identifying and quantifying cell phenotypes. *Genome Biol* **7**, R100 (2006).
