# Supplementary material for: A deep learning-based stripe self-correction method for stitched microscopic images
Source: Nat Commun. 2023 Sep 5;14:5393. doi: 10.1038/s41467-023-41165-1 (PMC10480181; doi:10.1038/s41467-023-41165-1)
Supplement: Supplementary file 3 — Reporting Summary [file 41467_2023_41165_MOESM3_ESM.pdf]

nature portfolio

Corresponding author(s): Wenxi Liu, Feng Huang, and Jianxin Chen

Last updated by author(s): Jul 26, 2023

Reporting Summary

Nature Portfolio wishes to improve the reproducibility of the work that we publish. This form provides structure for consistency and transparency in reporting. For further information on Nature Portfolio policies, see our Editorial Policies and the Editorial Policy Checklist.

Statistics

For all statistical analyses, confirm that the following items are present in the figure legend, table legend, main text, or Methods section.

n/a	Confirmed	
☐	☒	The exact sample size (*n*) for each experimental group/condition, given as a discrete number and unit of measurement
☐	☒	A statement on whether measurements were taken from distinct samples or whether the same sample was measured repeatedly
☐	☒	The statistical test(s) used AND whether they are one- or two-sided<br>*Only common tests should be described solely by name; describe more complex techniques in the Methods section.*
☒	☐	A description of all covariates tested
☐	☒	A description of any assumptions or corrections, such as tests of normality and adjustment for multiple comparisons
☐	☒	A full description of the statistical parameters including central tendency (e.g. means) or other basic estimates (e.g. regression coefficient) AND variation (e.g. standard deviation) or associated estimates of uncertainty (e.g. confidence intervals)
☐	☒	For null hypothesis testing, the test statistic (e.g. *F*, *t*, *r*) with confidence intervals, effect sizes, degrees of freedom and *P* value noted<br>*Give P values as exact values whenever suitable.*
☒	☐	For Bayesian analysis, information on the choice of priors and Markov chain Monte Carlo settings
☒	☐	For hierarchical and complex designs, identification of the appropriate level for tests and full reporting of outcomes
☒	☐	Estimates of effect sizes (e.g. Cohen's *d*, Pearson's *r*), indicating how they were calculated

Our web collection on statistics for biologists contains articles on many of the points above.

Software and code

Policy information about availability of computer code

Data collection	MPM dataset were scanned via an imaging system that was built on a commercially laser scanning microscope platform (LSM 880 Zeiss, Germany) using a mode-locked femtosecond Ti:Sapphire laser (Chameleon ultra, Coherent, America). Fluorescence, SRS, and H&E datasets are publicly available datasets.
Data analysis	The pretrained model, code, and relevant resources of the proposed SCCOR are released in https://github.com/lxxcontinue/SSCOR. SSCOR was trained and implemented using Python version 3.7 with Pytorch version 1.7.1. The benchmark methods include CIDRE (https://github.com/smithk/cidre, released on Aug 2, 2021), BaSiC (https://github.com/marrlab/BaSiC, released on Jan 20, 2022), ZEN (https://www.zeiss.com/microscopy/en/products/software/zeiss-zen.html), ZeroDCE (https://github.com/Li-Chongyi/Zero-DCE, released on Mar 9, 2022), Neighbor2Neighbor (https://github.com/TaoHuang2018/Neighbor2Neighbor, released on Sep 22, 2021), and Mask-shadowGAN (https://github.com/xw-hu/Mask-ShadowGAN, released on Aug 12, 2021). The correction quality was evaluated by a user study and the following metrics. Inverse coefficient variation (ICV) was calculated using Python version 3.9.5. The full-reference quality metrics (PSNR and SSIM) were calculated using Python version 3.9.5. The contrast of collagen fibers (Fig. 5) was calculated using Matlab vR2021b (The MathWorks Inc.). Virtual staining was achieved by the open-source model UTOM (https://github.com/cabooster/UTOM, released Jun 9, 2022). Cell segmentation and classification were performed by the open-source Hover-Net (https://github.com/vqdang/hover_net, released on Dec 23, 2022). The cell numbers were counted using the software package CellProfiler v4.2.1 (https://github.com/CellProfiler/CellProfiler). The number and area of collagen fibers were extracted and qualified by CurveAlign v4.0 (https://eliceirilab.org/software/curvealign/).

For manuscripts utilizing custom algorithms or software that are central to the research but not yet described in published literature, software must be made available to editors and reviewers. We strongly encourage code deposition in a community repository (e.g. GitHub). See the Nature Portfolio guidelines for submitting code & software for further information.

Data

Policy information about availability of data

All manuscripts must include a data availability statement. This statement should provide the following information, where applicable:

- Accession codes, unique identifiers, or web links for publicly available datasets
- A description of any restrictions on data availability
- For clinical datasets or third party data, please ensure that the statement adheres to our policy

The MPM data of non-uniform/grid stripes used in this study are available at our github repository https://github.com/lxxcontinue/SSCOR. The fluorescence dataset used for oblique stripes are available at http://brainarchitecture.org/. The SRS dataset used for special artifacts synthesis are available at https://dataverse.harvard.edu/dataset.xhtml?persistentId=doi:10.7910/DVN/EZW4EK. The CoNSep dataset used for stripes synthesis are available at https://warwick.ac.uk/fac/sci/dcs/research/tia/data/. The MoNuSAC dataset used for stripes synthesis are available at https://monusac-2020.grand-challenge.org/Data/. Source data are provided with this paper and our github repository.

Human research participants

Policy information about studies involving human research participants and Sex and Gender in Research.

Reporting on sex and gender	*Use the terms sex (biological attribute) and gender (shaped by social and cultural circumstances) carefully in order to avoid confusing both terms. Indicate if findings apply to only one sex or gender; describe whether sex and gender were considered in study design whether sex and/or gender was determined based on self-reporting or assigned and methods used. Provide in the source data disaggregated sex and gender data where this information has been collected, and consent has been obtained for sharing of individual-level data; provide overall numbers in this Reporting Summary. Please state if this information has not been collected. Report sex- and gender-based analyses where performed, justify reasons for lack of sex- and gender-based analysis.*
Population characteristics	*Describe the covariate-relevant population characteristics of the human research participants (e.g. age, genotypic information, past and current diagnosis and treatment categories). If you filled out the behavioural & social sciences study design questions and have nothing to add here, write "See above."*
Recruitment	*Describe how participants were recruited. Outline any potential self-selection bias or other biases that may be present and how these are likely to impact results.*
Ethics oversight	*Identify the organization(s) that approved the study protocol.*

Note that full information on the approval of the study protocol must also be provided in the manuscript.

Field-specific reporting

Please select the one below that is the best fit for your research. If you are not sure, read the appropriate sections before making your selection.

- [x] Life sciences
- [ ] Behavioural & social sciences
- [ ] Ecological, evolutionary & environmental sciences

For a reference copy of the document with all sections, see nature.com/documents/nr-reporting-summary-flat.pdf

Life sciences study design

All studies must disclose on these points even when the disclosure is negative.

Sample size	To demonstrate the generalization ability of SSCOR, this study collected four stitched microscopic datasets across different imaging conditions and modalities. The sample size comprised forty images with various stripes or artifacts in these datasets, including eighteen H&E images (used for synthesizing stripes), fifteen MPM images, five fluorescence images, and two SRS images (used for synthesizing stripes, scanning, bubble, and out-of-focus artifacts, resulting in five synthetic images). The resolution of each image ranges from 1000×1000 pixels to 7350x5390 pixels, providing sufficient unpaired patches for the unsupervised model training and validation. No statistical method was used to predetermine sample size.
Data exclusions	No data were excluded from the analyses.
Replication	Under the unsupervised learning settings, for each input image, all the models were trained once per set of hyper-parameters, and tested on the same image. With different initial weights, the proposed SCCOR eventually achieved similar results. To facilitate reproduction, we release the trained model, codes, and source data at https://github.com/lxxcontinue/SSCOR.
Randomization	The randomization of sample allocation is irrelevant to our study, since no train-test split is applied in the unsupervised setting of SSCOR. The proposed method achieves stripe correction by sampling training patches directly from the test image itself without accessing ground truths. To evaluate SSCOR, all the comparison methods and the proposed method were trained and tested under the same protocol without any randomization.

Blinding	Due to the inaccessibility of the ground-truths in practical situations, the correction process was blinded to the ground-truths during training and inference.

Reporting for specific materials, systems and methods

We require information from authors about some types of materials, experimental systems and methods used in many studies. Here, indicate whether each material, system or method listed is relevant to your study. If you are not sure if a list item applies to your research, read the appropriate section before selecting a response.

Materials & experimental systems

n/a	Involved in the study	
☒	☐	Antibodies
☒	☐	Eukaryotic cell lines
☒	☐	Palaeontology and archaeology
☒	☐	Animals and other organisms
☒	☐	Clinical data
☒	☐	Dual use research of concern

Methods

n/a	Involved in the study	
☒	☐	ChIP-seq
☒	☐	Flow cytometry
☒	☐	MRI-based neuroimaging
